# Novel scaffolds for inhibition of Cruzipain identified from high-throughput screening of anti-kinetoplastid chemical boxes

**DOI:** 10.1038/s41598-017-12170-4

**Published:** 2017-09-21

**Authors:** Emir Salas-Sarduy, Lionel Urán Landaburu, Joel Karpiak, Kevin P. Madauss, Juan José Cazzulo, Fernán Agüero, Vanina Eder Alvarez

**Affiliations:** 1 Instituto de Investigaciones Biotecnológicas – Instituto Tecnológico de Chascomús, Universidad Nacional de San Martín – CONICET, San Martin, B1650HMP Buenos Aires Argentina; 2 GlaxoSmithKline R&D, Molecular Design US, Pennsylvania, Upper Providence PA USA; 3 GlaxoSmithKline R&D, Trust in Science, Pennsylvania, Upper Providence PA USA

## Abstract

American Trypanosomiasis or Chagas disease is a prevalent, neglected and serious debilitating illness caused by the kinetoplastid protozoan parasite *Trypanosoma cruzi*. The current chemotherapy is limited only to nifurtimox and benznidazole, two drugs that have poor efficacy in the chronic phase and are rather toxic. In this scenario, more efficacious and safer drugs, preferentially acting through a different mechanism of action and directed against novel targets, are particularly welcome. Cruzipain, the main papain-like cysteine peptidase of *T. cruzi*, is an important virulence factor and a chemotherapeutic target with excellent pre-clinical validation evidence. Here, we present the identification of new Cruzipain inhibitory scaffolds within the GlaxoSmithKline HAT (Human African Trypanosomiasis) and Chagas chemical boxes, two collections grouping 404 non-cytotoxic compounds with high antiparasitic potency, drug-likeness, structural diversity and scientific novelty. We have adapted a continuous enzymatic assay to a medium-throughput format and carried out a primary screening of both collections, followed by construction and analysis of dose-response curves of the most promising hits. Using the identified compounds as a starting point a substructure directed search against CHEMBL Database revealed plausible common scaffolds while docking experiments predicted binding poses and specific interactions between Cruzipain and the novel inhibitors.

## Introduction

American Trypanosomiasis or Chagas Disease is a prevalent, neglected, debilitating and potentially life-threatening tropical disease caused by the kinetoplastid protozoan parasite *Trypanosoma cruzi*^1^. The disease is the leading cause of congestive heart failure in Latin America and cause 20,000 deaths every year, thus representing a substantial social and economic burden. Chagas disease is a vector-borne disease but can also be transmitted congenitally, by way of blood transfusions or organ transplants. Owing to the extensive global migration of asymptomatic, chronically infected individuals from endemic regions, Chagas disease now affects thousands of people in nonendemic regions like North America and Europe^2^. The current chemotherapy is limited only to nifurtimox and benznidazole, two drugs associated with long term treatments, reduced efficacy during the chronic phase and severe side effects^1^. In this scenario, more efficacious and safer drugs, especially those acting through a different mechanism of action and directed against novel targets, are particularly welcome.

**Figure 1:**
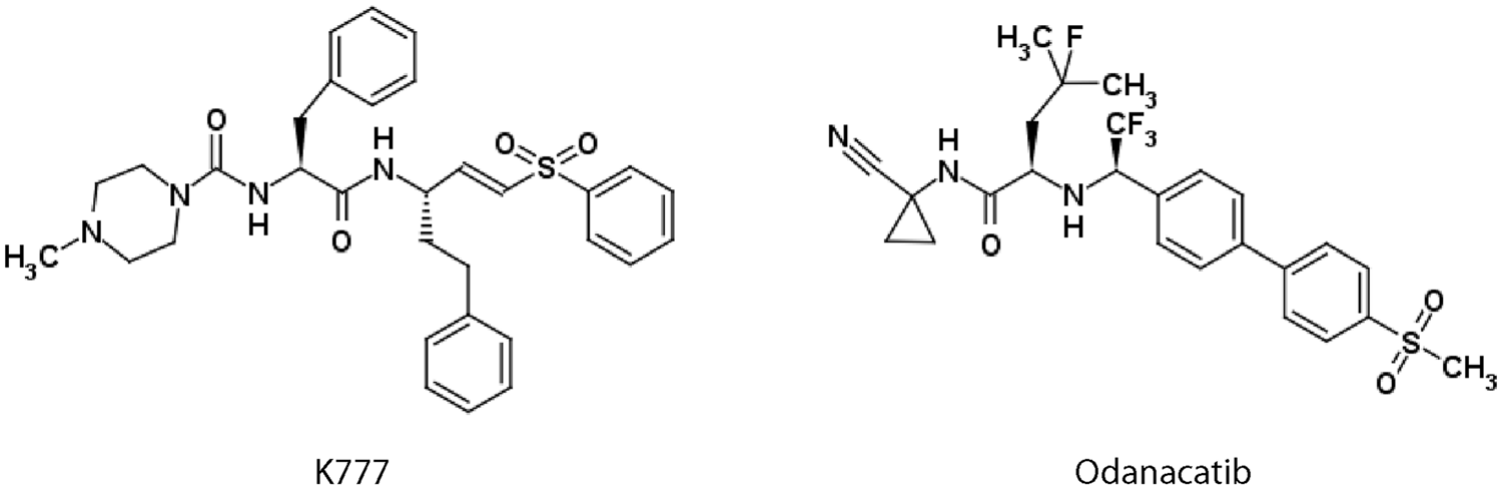
Structures of K777 and Odanacatib.

Cruzipain (EC 3.4.22.51), belonging to Clan CA family C1, is the main cysteine peptidase of *T. cruzi*^3^. It is produced by a large family of polymorphic genes encoding highly similar isoforms (approximately 88–98% amino acid identity)^4^, which are globally responsible for the majority of the proteolytic activity present in epimastigotes. Cruzipain is expressed in the four main stages of *T. cruzi* life cycle^5^, and is present in lysosome-related organelles^6^. The highest concentration is found in epimastigote-specific pre-lysosomal organelles called reservosomes^7,8^, although it could also be associated to plasma membrane in amastigotes^9^ and secreted to the extracellular medium by trypomastigotes^10^. Cruzipain is an important virulence factor and a chemotherapeutic target with excellent pre-clinical validation evidence. It is involved in parasite nutrition, invasion of mammalian cells and evasion of the host immune response^11,12^. In addition, Cruzipain participates in differentiation steps of the parasite’s life cycle, which are blocked by permeant irreversible inhibitors of the enzyme^13^ and enhanced by its over-expression^14^. Specific inhibitors of this enzyme effectively inhibit parasite invasion and replication in mammalian cells^13,15,16^, as well as promote direct parasite killing in culture^17^. In this regard, the irreversible vinyl sulfone Cruzipain inhibitor K777 (N-[(2 S)-1-[[(E,3 S)-1-(benzenesulfonyl)-5-phenylpent-1-en-3-yl]amino]-1-oxo-3-phenylpropan-2-yl]-4-methylpiperazine-1-carboxamide; Fig. 1) has been efficacious in preclinical models of *T. cruzi* infection, including immunocompetent and immunodeficient mice^18,19^ and dogs^20^. Inspired by Odanacatib (Fig. 1), a reversible Cathepsin K inhibitor containing a nitrile “warhead”, several Cruzipain inhibitors were also identified by Merck in a drug discovery effort and further refined^21^, resulting in nanomolar, covalent but yet reversible Cruzipain inhibitors with moderate antiparasitic activity both *in vitro* and *in vivo*^22,23^.

Using as starting point the 1,8 million GlaxoSmithKline HTS collection, three anti-kinetoplastid chemical boxes, grouping around 200 compounds each, were recently assembled after an orthogonal phenotypic screening against *L. donovani*, *T. brucei* and *T. cruzi*^24^. This selection was aimed to maximize antiparasitic potency, drug-likeness, structural diversity and scientific novelty to minimize non-specific cellular cytotoxicity and fuel the identification of new target-specific antiparasitic scaffolds, not related to those in current clinical use. Diverse (hypothetical) modes of actions were computationally predicted for the compounds included in the chemical boxes and, interestingly, several cysteine peptidases, including those from Clan CA family C1, were identified as their putative targets.

In this paper, we report the identification of Cruzipain inhibitors within the GSK HAT (Human African Trypanosomiasis) and Chagas chemical boxes. To this end, we have adapted a continuous enzymatic assay to a medium-throughput format and carried out a primary screening of both collections, followed by construction and analysis of dose-response curves of the most promising hits. Using the identified compounds as a starting point a substructure directed search against CHEMBL Database^25^ revealed plausible common scaffolds while docking experiments predicted binding poses and specific interactions between Cruzipain and the novel inhibitors.

## Results

### Development of a continuous Cruzipain Assay

With the aim to evaluate the GSK HAT and Chagas Chemical boxes, a continuous fluorogenic Cruzipain assay was developed, using Z-FR-AMC as substrate^26^. The optimization process was carried out in 384-well plates, the same format that has been used for the screening of the chemical boxes. To determine a convenient Cruzipain concentration for the assay, the activity of 2-fold serial enzyme dilutions was assayed at a fix substrate concentration of 2 µM, previously reported as the K_M_ value for the system under study^27^. For [Cruzipain]_0_ ≤ 29.4 nM, progression curves remained linear for at least 60 minutes (Supplementary Figure S1A) and the Selwyn test^28^ indicated that enzyme remained stable during the assay (Supplementary Figure S1B). For a wide range of Cruzipain concentrations, the V_0_ vs. [E]_0_ curve showed the expected linear behavior (Supplementary Figure S1C) and neither Triton X-100 (0–0.03%) nor DMSO (0–3%) induced appreciable changes in Cruzipain activity (not shown). At [Cruzipain]_0 _= 5.8 nM, a proper balance was observed between Cruzipain activity (estimated as dF/dt) and the time over which the reaction displayed linear kinetics. Under these conditions, the enzyme showed the typical hyperbolic behavior predicted by the Michaelis-Menten equation (Hill coefficient = 1.02 ± 0.05; Supplementary Figure S1D,E) and an estimated K_M_ value of 1.52 ± 0.14 µM, very similar to that previously reported^27^. To avoid biases in the inhibition mechanisms of hits during the screening^29^, substrate concentration in the assay was fixed at 2 µM (~1.3 K_M_). In the absence of Cruzipain, negligible or no spontaneous hydrolysis was observed for Z-FR-AMC substrate; although some level of photo bleaching was suggested by the linear decay in Fluorescent readouts with time. During preliminary characterization experiments the optimized assay showed good general performance, with a dynamic range (µ^C+^ − µ^C−^) higher than 800 RFU/sec, a µ^C+^/µ^C−^ ratio ≥ 260, good reproducibility (VC < 5%) and a Z’ factor^30^ value around 0.9.

**Figure 2:**
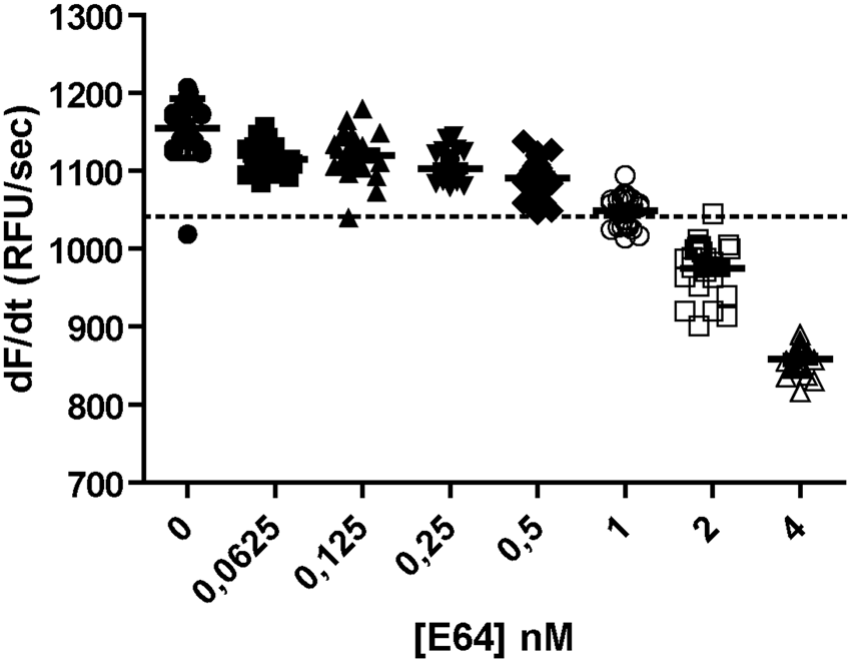
Determination of inhibitor detection limit for the optimized Cruzipain assay. Different E-64 concentrations were assessed to determine the inhibitor concentration causing an average inhibition equivalent to the statistically robust cutoff ([*I*]^MINIMAL^). For clarity, only E-64 concentrations ranging from 0 (enzyme control) to 4 nM are shown. To accurately describe the data dispersion, 24 replicates were used for each concentration point.

Once we established these experimental conditions, we next tested the ability of the developed assay to detect the inhibition caused by decreasing concentrations (10 µM–0.0625 nM) of E-64, an irreversible inhibitor of Clan CA cysteine peptidases (Fig. 2). At 1 nM ([E-64]/[Cruzipain] ≈ 0.17), the average slope of the inhibited reaction was equivalent to the statistically robust cutoff (µ^C+^ − 3 × SD^C+^), which corresponded to an average 9.87% inhibition with respect to Cruzipain control. Therefore, this E-64 concentration represents the lower limit for detectability of high potent inhibitors (i.e. tight-binding inhibitors; Ki ^app^ ≤ 10^−7^ M) in the developed assay. At this concentration, even for this potent and low-variable inhibition rates, nearly half the E-64 replicates were above the established cutoff, resulting in negative samples if analyzed individually. As observed in Fig. 2, a significantly higher inhibitor concentration is further required in practice to achieve 100% of positive replicates. For less potent inhibitors (i.e. classical inhibitors; Ki ^app^ ≥ 10^−6^ M), the minimum compound concentration required to reach the limit of 9.87% Cruzipain inhibition, and thus needed to be detected by the assay, is expected to be significantly higher. Given that we anticipate a theoretical detection limit of 0.11 µM for compounds showing Ki^app^ ≥ 10^−6^ M under assay conditions (see Methods for deduction), we decided to use a fix compound concentration of 25 µM during primary screening to give enough margin for compounds with low potency and high variability to be detected in singlet measurements.

### Primary screening of HAT and Chagas chemical boxes

The 404 compounds present in the HAT and Chagas chemical boxes were screened in singlet at a fixed dose of 25 µM ([Inhibitor]/[Cruzipain] ≈ 4310; expected Ki ^app^ ≤ 228 µM), using the same batch of substrate and enzyme in a lapse of three hours. In addition to the compounds (plate 1: 320; plate 2: 84), both plates included enzyme (n = 24), substrate (n = 24) and inhibition (n = 16) controls alternately located in columns 11, 12, 23 and 24. Although slightly different (presumably due to automatic re-scaling of Fluorescent readouts), the resulting Z´ factor values for the plates were rather high (0.92 and 0.68) during the screening. Statistics for each plate are resumed in Table 1.

**Table 1:** Statistics for the plates during primary screening.

	Plate 1		Plate 2	
	Mean	SD	Mean	SD
Cruzipain Control (C^+^) (RFU/sec)	893.31	23.35	210.84	22.29
Negative Control (C^−^)(RFU/sec)	−2.68	1.44	0.80	0.13
E-64 Control (RFU/sec)	19.30	4.84	6.36	0.50
Compounds (RFU/sec)	794.91	135.38	191.89	46.74
Compounds Inhibition (%)	11.03	15.11	9.02	22.25
Z factor	0.917		0.679	
Statistically robust cutoff (RFU/sec)	823.26		143.97	
Hit threshold i (RFU/sec)	388.77		51.67	
Hit threshold ii (%)	56.36		75.77	
Autofluorescence cutoff (RFU)	972818.33		229605.97	

**Figure 3:**
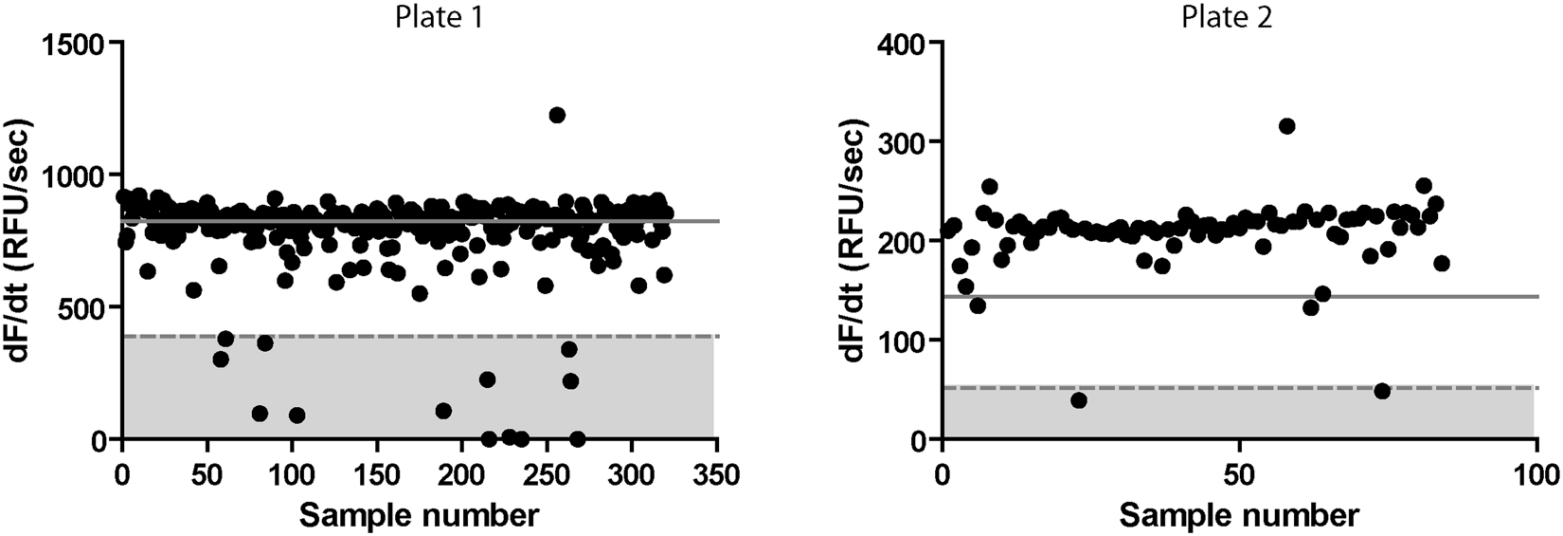
Activity slopes for the compounds in HAT and Chagas chemical boxes during primary screening against Cruzipain. The solid line shows the statistically robust cutoff; the dashed line shows the cutoff for selection of outliers (selection criteria i, see main text).

After screening, 158 compounds (~39%) showed experimental slope values under the statistical robust cutoff (µ^C+^ − 3 × SD^C+^) (Fig. 3). Given the high number of resultant hits, two more stringent selection criteria, focusing only in outliers, were adopted: i) those compounds showing slopes >3 standard deviations below the average of all slopes in the plate (control independent) and ii) those compounds showing a percent inhibition >3 standard deviations above the average for the plate (control dependent). Interestingly, both criteria retrieved exactly the same list of hit compounds (total: 15; plate 1: 13; plate 2: 2), hence resulting equivalent despite differences in data pre-processing (normalization), control dependency and statistical robustness. At the excitation/emission wavelengths used for AMC recording, 26 compounds (plate 1: 19; plate 2: 7) showed significant auto-fluorescence in the absence of substrate (Supplementary Figure S2), including 7 present in the reduced hit list. Despite the negative impact in reproducibility associated to the inclusion of highly fluorescent compounds in hit selection^31,32^, we decided not to exclude *a priori* any compound and to move forward with the complete hit list to the secondary screening.

### Secondary screening

**Figure 4:**
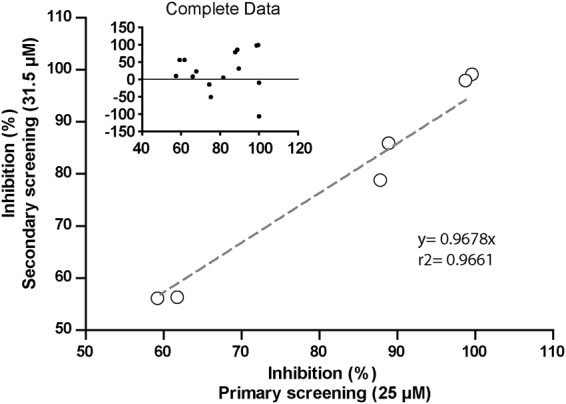
Correlation between inhibition percentages in the primary and secondary screenings for compounds in group 1. Analysis was performed using a single concentration of 25 µM and 31.5 µM for primary and secondary screening, respectively. The inset shows a correlation analysis for all the hit compounds at indicated concentrations.

To estimate IC_50_ values for the resulting 15 hits from the primary screening, two-fold serial dilutions, ranging from 62.5 µM to 7.5 pM, were analyzed against Cruzipain using the developed assay. Enzyme concentration was doubled (11.6 nM) to increase differences between classical and tight-binding inhibitors, given that several compounds have produced virtually total inhibition during primary screening. Prior to the analysis of the complete dataset, we looked for correlations between inhibition percentages in the primary (25 µM) and secondary screening, using only data corresponding to a compound concentration of 31.5 µM. Figure 4 show that 6 compounds exhibited consistent results in both screenings (group 1, correlation coefficient r^2^ = 0.966; slope = 0.967), 5 failed to repeat inhibition (group 2, correlation coefficient r^2^ = 0.219; slope = 0.1725) and 4 compounds displayed Cruzipain activation instead of inhibition in the second round (group 3, correlation coefficient r^2^ = −0.5291; slope = 0.0847) (Supplementary Figure S3).

With only two exceptions, those compounds that failed to repeat inhibition (groups 2 and 3) were among those previously identified as auto fluorescent in the primary screening. This auto fluorescence was further confirmed by the observation that progression curves for different concentrations started at very different initial fluorescence readouts, decreasing with compound dilution (Supplementary Figure S4C–E). Given that the contribution of Cruzipain-hydrolyzed AMC to the total fluorescence in these cases is usually low; the associated error in dF/dt determination is high enough to result in erratic dose-response curves (Supplementary Figure S4D–F). In addition, at the higher concentrations tested, it would be possible that the occurrence of complex compound-specific phenomena such as the formation of colloidal aggregates may have interfered with fluorescence measurements or generated an interaction surface with Cruzipain artificially increasing or decreasing its activity.

**Figure 5:**
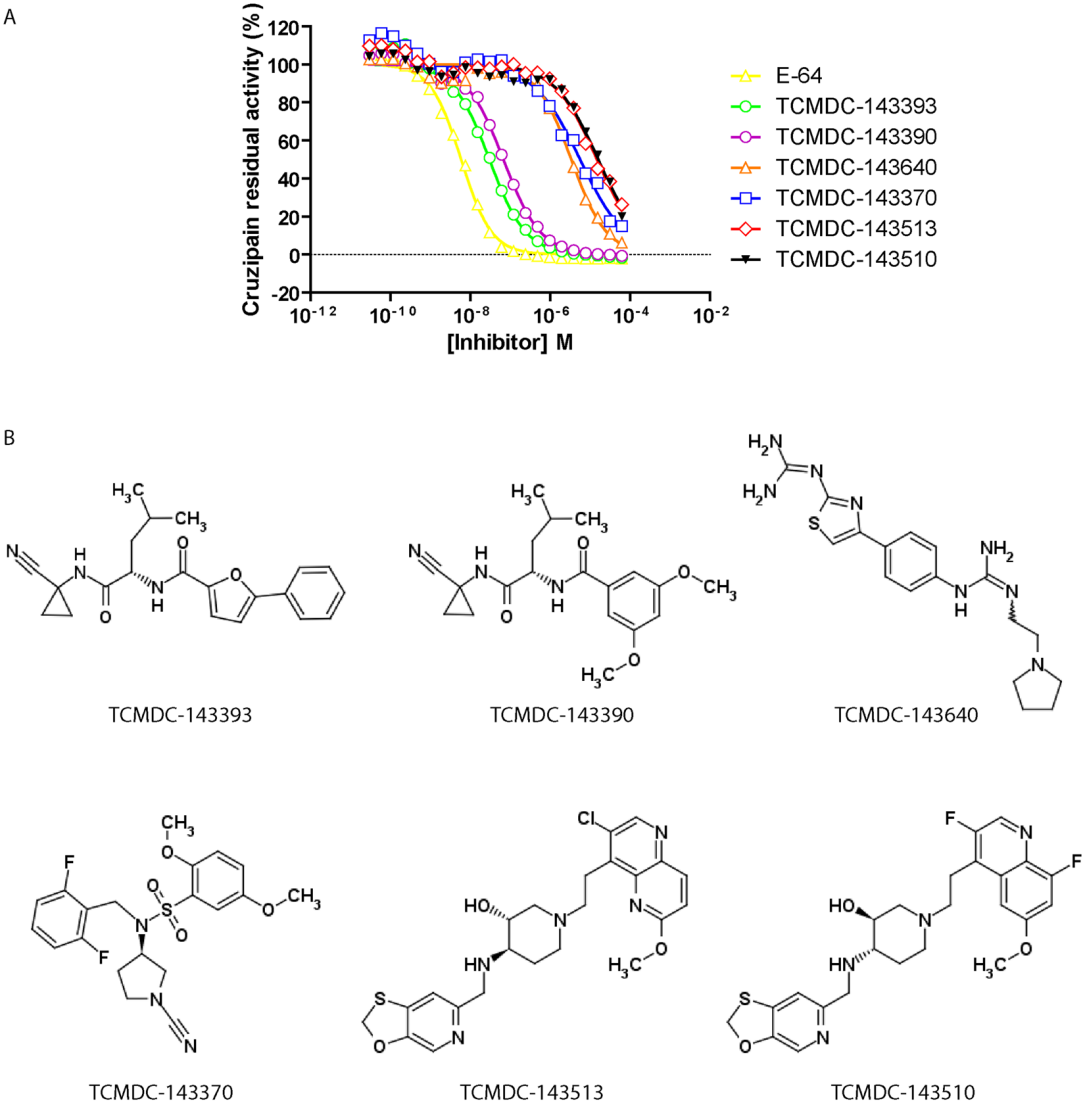
Dose-response curves and structures of identified Cruzipain inhibitors. (**A**) For each compound, solid line represents the best fit of four-parameter Hill equation to experimental data (open/closed figures). (**B**) Structure and identifiers corresponding to the hit compounds identified.

In contrast, progression curves from group 1 compounds showed the expected behavior, with similar initial fluorescence readouts for all dilutions (Supplementary Figure S4A,B). The fitness of dose-response curves to the four-parameter model was good (even for incomplete curves) and the values for Hill coefficient around -1 were in accordance with the expected 1:1 enzyme/compound interaction (Table 2). Importantly, the validated hits were diverse both in structure (Fig. 5) and potency, with apparent IC_50_ values in the micromolar and submicromolar range.

**Table 2:** IC_50_ values and Hill slopes for the identified hits.

Compound	Chemical Box	IC_50_ μM	Hill Slope	R square
E-64	—	0,005937	−1,137	0,9961
TCMDC-143393	HAT	0,03025	−0,957	0,9899
TCMDC-143390	HAT	0,06783	−0,9354	0,996
TCMDC-143640	HAT	3,393	−0,9825	0,9848
TCMDC-143370	HAT	5,646	−0,7542	0,9516
TCMDC-143513	HAT	14,94	−0,8108	0,9626
TCMDC-143510	HAT	16,15	−0,7998	0,9627

### Chemical similarity amongst compounds in GSK Chagas and HAT boxes

To see if the screened Chagas and HAT chemical boxes contained other compounds with chemical similarity to the identified hits, we performed “*all vs all”* fingerprint-based similarity searches using the Tanimoto index as the similarity metric, focusing specifically in positive hits obtained in the *in vitro* screening. Besides the obvious similarity between some the identified hit compounds (e.g. the pairs TCMDC-143390/TCMDC-143393 or TCMDC-143510/TCMDC-143513, see Fig. 5), no other significant similarities were found, other than TCMDC-143395 which was predicted to be a similar compound to the 143390/143393 pair but was absent in the *in vitro* screening collection provided by GSK. Results on Tanimoto index screening are available in Supplementary Figure S5.

### Scaffold determination through computational methods

**Figure 6:**
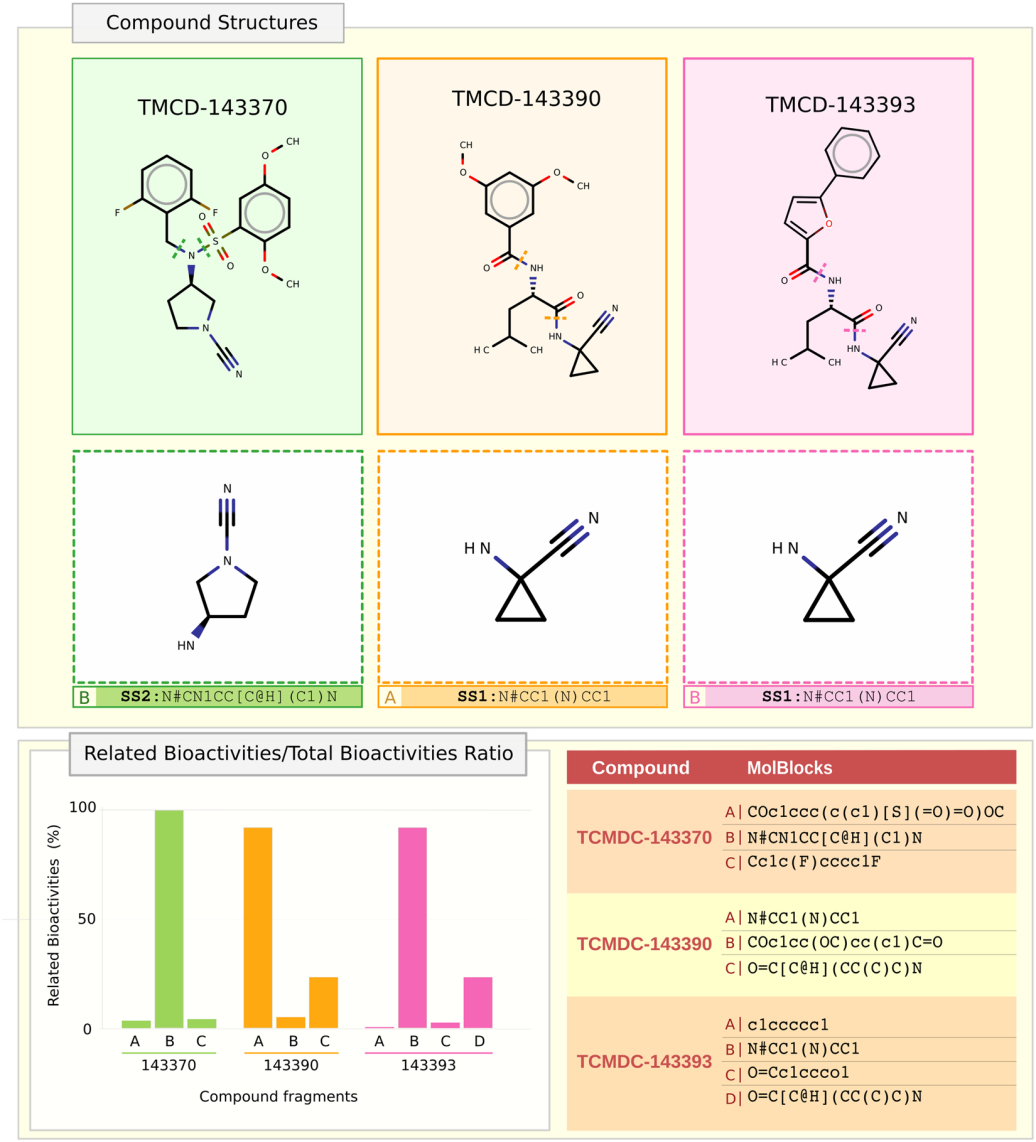
Fragment Substructures enriched in Protease Bioactivities. Substructures from active hits against Cruzipain were obtained using MolBlocks: (**A**) Structures and selected molblocks for the Cruzipain active hits identified in this work; (**B**) Bar chart showing enrichment in activity against protease targets in ChEMBL. The complete list of fragments obtained for every molecule in SMARTS notation. Related Bioactivities/Total Bioactivities Ratio: Comparison between bioactivities found for every molecule in the substructure fragment search.

Starting from the chemical structures of the identified Cruzipain inhibitors, several molecular fragments were obtained using MolBlocks^33^ (Fig. 6). All fragments were used as queries in substructure searches against the ChEMBL database (Release 22.1) to retrieve structurally related bioactive compounds. These compounds were then used to search for all reported single-target bioactivities in this database. The identified bioactivities were finally filtered by assessing whether the assayed target was a protease or not. Then, a simple ratio was calculated as the quotient between the number of related bioactivities (target is a protease) and the number of total bioactivities reported. Figure 6 (and Supplementary Figure S7) show these ratios as percentages.

From these results it is clear that at least two scaffolds associated with protease inhibition can be proposed according to the currently available data: Substructure Scaffold 1 (SS1), represented by Fragment A from TCMDC-143390, and fragment B from TCMDC-143393; and a substructure Scaffold 2 (SS2), represented by fragment B from TCMDC-143370. For SS1, 374 drug hits were retrieved from a substructure search vs ChEMBL, for which a total amount of 1,952 single-target bioactivities could be associated, 887 of which were reported for proteases. In the case of SS2, a total of 23 drug hits were found in these searches, retrieving 171 single-target bioactivities (66 of which were against proteases). All compound fragments and raw data for the calculations are available as Supplementary Material (Figures S6 and S7, respectively).

### Docking of hit compounds on Cruzipain active site and prediction of binding pose

**Figure 7:**
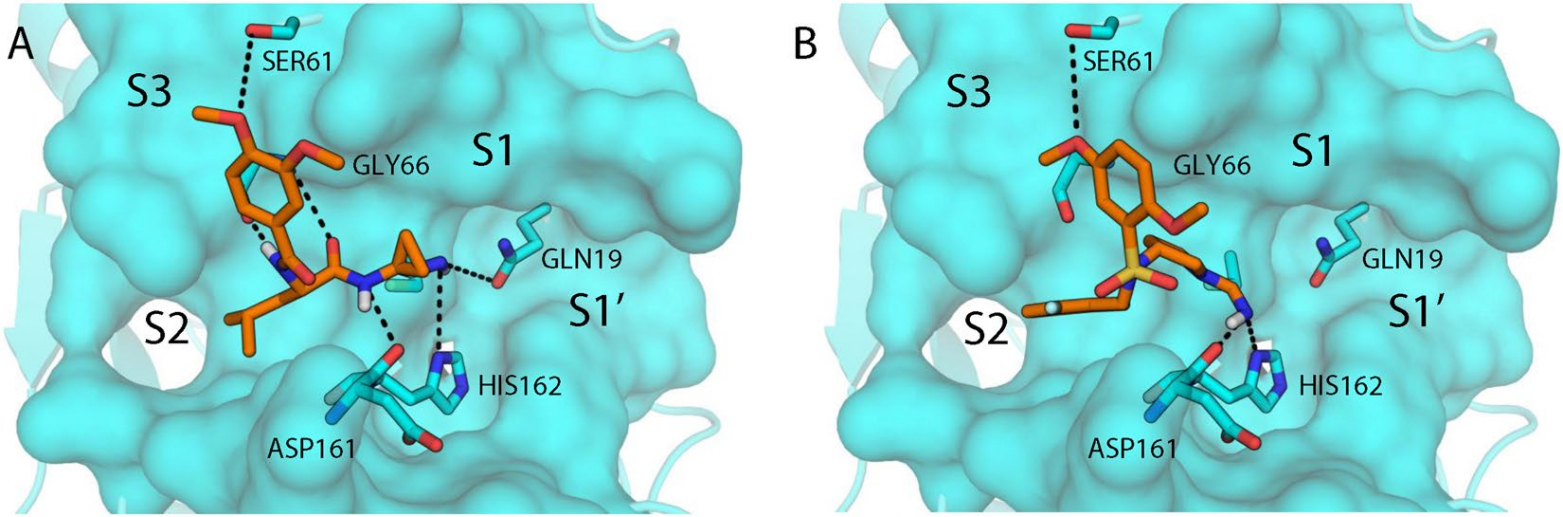
Docked poses of TCMDC-143390 (**A**, orange) and TCMDC-143370 (**B**, orange) to Cruzipain (PDB: 3IUT, cyan). Putative hydrogen bonds are shown as dashed black lines, illustrating the similar hydrophobic and electrostatic interactions in each labeled subsite between the discovered inhibitors.

After the identification of SS1 and SS2 as protease inhibitor scaffolds with putative nitrile warheads, we used the covalent docking protocol CovDock^34^ in order to investigate the specific interactions of representatives from each substructure with Cruzipain (PDB: 3IUT). We defined Cys25, the known nucleophile for other covalent inhibitors, as the covalent anchor point and docked both TCMDC-143390 and TCMDC-143370 as attached thioimidates (Fig. 7). Like other known peptidic and nonpeptidic inhibitors, both compounds extend into the S2 and S3 subsites, yet they retain potency without filling the S1 or S1’ subsites or the oxyanion hole. The isopropyl group of TCMDC-143390 and the difluorobenzyl group of TCMDC-143370 provide pocket-filling hydrophobic interactions in the S2 subsite between Leu67 and Leu160. Another parallel between SS1 and SS2 is the role of the pyrrolidine and cyclopropyl groups, which act as conformational stabilizers and provide the trajectories to fill the other subsites. The S3 pocket is filled with similar dimethoxyphenyl moieties, which use one of the oxygens as a hydrogen bond acceptor to Ser61. However, unlike TCMDC-143390, which canonically uses both amides as hydrogen bond acceptors and donors to the backbone amide of Gly66, TCMDC-143370 manages to achieve the same overall conformation through the combination of the saturated pyrrolidine and sulfonamide. The sulfonamide does not make any polar interactions with the enzyme, and the sulfone oxygens are likely stabilized by solvent water molecules. These differences suggest that TCMDC-143370 represents a novel Cruzipain binding motif that can be exploited for further analog design.

## Discussion

Scaffold 1 is characterized by the presence of a nitrile warhead bound to the C1 of cyclopropyl amide derivatives. It is represented in the anti-kinetoplastid boxes by TCMDC-143393 and TCMDC-143390 showing IC_50_ values in the submicromolar range against Cruzipain. Interestingly, both IC_50_ values are in the same order of the enzyme concentration used in the assay. This observation strongly suggests that both compounds may constitute tight-binding Cruzipain inhibitors with moderate potency (Ki ≈ 1 to 4 × 10^−8^ M). The close structural resemblance among the compounds also suggests that the slight differences observed in potency would be related with differential accommodation of the aromatic rings substituting the main structure into Cruzipain subsites. Furthermore, the structural similarities shared by these compounds with Odanacatib (Fig. 1), a potent Cathepsin K inhibitor, or with other nanomolar Cruzipain inhibitors which form a reversible thioimidate with the active site cysteine of their target enzymes^35,36^, also suggest a similar (competitive) inhibition mechanism for the identified compounds. This scaffold was predicted as one of the three chemotypes active against cysteine peptidases within the chemical boxes^24^ with three predicted compounds TCMDC-143390 (HAT), TCMDC-143393 (HAT) and TCMDC-143395 (missing in our HAT collection, *in silico*); all of them identified by us in this study.

We would like to point out, that at least one of them (TCMDC-143393) has in fact a reported bioactivity against *T. cruzi*; however since IC50 values are between one and two orders of magnitude higher than those obtained from *T. brucei*, this compound was only included in the HAT box, because it did not meet the selection criteria used to build up the Chagas box. Considering that the developed growth inhibition assays were performed on axenic bloodstream forms in the case of *T. brucei* but on intracellular amastigotes in the case of *T. cruzi*, some differences on IC50 values may be anticipated on the basis of the number of membranes that have to be crossed by the compunds.

In previous studies, the presence of a nitrile warhead has been described as a problematic functional group due to its ability to react with S^−^ residues in proteins, leading to potential pan-assay interferences^37^. Many nitrile-containing compounds, however, have shown to be non-promiscuous inhibitors of cysteine peptidases^21,38,39^. In this regard, the identified compounds exhibit relatively low inhibition frequency indexes (2.04, 4.08 and 2.06, respectively) in comparison with the distribution of this parameter within the compounds in the chemical boxes (Figure S8), suggesting that inter-assay selectivity would not be an issue. Currently, no estimations are available for the off-rates of these compounds from Cruzipain-Inhibitor complexes. Even in the case of significantly low off-rate values (i.e. indicating irreversible binding to the target for all practical purposes), several examples of this class of compounds are currently in late stages of clinical development^40^. Many others, including aspirin (cyclooxigenase inhibitor), penicillin antibiotics (bacterial DD-transpeptidase), clopidogrel (P2Y purinergic receptor 12), rasagiline and selegiline (monoamine oxidase inhibitors) and the proton pump inhibitors omeprazole, lansoprazole and esomeprazole, are largely prescribed medicines used for decades for different indications (including long-term therapies) with excellent efficacy and safety records^40^, clearly indicating that covalent irreversible inhibitors can be extremely effective when used in an appropriate therapeutic context^41^.

Scaffold 2 is represented by 2 compounds: TCMDC-143370 (HAT) and TCMDC-143238 (GSK LEISH chemical box, identified *in silico*), with TCMDC-143370 showing an IC_50_ value in the low micromolar range. Although the presence of a nitrile moiety might also stand for a covalent inhibition mechanism as previously discussed for Scaffold 1, both scaffolds show major structural differences. In this scaffold, the nitrile group is bound directly to the nitrogen atom of the pyrrolidine cycle, which is substituted in position 2 by an amine group with multiple substituents. As in the previous case, this scaffold has been predicted as active against cysteine peptidases^24^. Despite the common presence of the nitrile warhead, the compounds in this group show divergent values for the inhibition frequency index parameter (2.41 and 5.10 respectively) indicating the impact of substituent groups in the general reactivity of the compounds bearing this scaffold.

In contrast, TCMDC-143513 and TCMDC-143510 (see Fig. 5) showed very similar structures and potencies against Cruzipain, representing an entirely different scaffold. The computational search retrieved six compounds with similarity against this pair: TCMDC-143158 (HAT), TCMDC-143373 (HAT), TCMDC-143527(CHAGAS), TCMDC-143162 (CHAGAS), TCMDC-143514 (LEISH, *in silico*) and TCMDC-143538 (LEISH, *in silico*). All of the four compounds from the Chagas and HAT chemboxes proved to be positive hits using the less-stringent statistical robust cut-off used initially in this study, and one of them was also included in the secondary screening (group 2, highly autofluorescent). However, the fragment (molblock) computational analysis failed to retrieve smaller shared common substructures with known protease active compounds. Therefore, currently these seem to be the smallest structures representative of this family that are capable of inhibiting Cruzipain. Furthermore, and to the best of our knowledge, this is the first report of Cruzipain inhibitors with these scaffolds.

Regarding the putative binding modes of representative examples of SS1 and SS2 to the catalytic domain of Cruzipain, the covalent docking studies show good overall agreement with known inhibitor binding motifs. While TCMDC-143390 (SS1) exhibits many of the canonical interactions between the peptidic inhibitor and the protease, TCMDC-143370 (SS2) retains potency without many of the anchoring polar interactions present. By utilizing the novel core of the pyrrolidine and sulfonamide, TCMDC-143370 provides an alternative to substrate-mimicking peptidic inhibitors, and the docked model offers attractive starting points for designed optimization into each of the subpockets.

Altogether, our findings highlight the importance of the anti-kinetoplastid chemical boxes as a prominent source of novel molecules with the potential to become drug candidates against well-validated proteolytic targets. In this regard, it seems important to keep the screening as wide as possible (i.e. not to circumscribe the study just to the chemical box specific for the species for which the target enzyme is from), as unique compounds representing promising scaffolds not present in other boxes might be missed.

Due to its versatility, the MTS methodology used here have been successfully extrapolated to other proteases validated as promising drug targets for kinetoplastid parasites. Given their potency and/or novelty, the inhibitory chemotypes identified in this study represent promissory starting points for future hit–to-lead optimization. Most important, we plan to extend the study to other compound bearing the proposed scaffolds from the GlaxoSmithKline chemical diversity, in order to expand in the near future our therapeutic arsenal against Chagas disease.

## Methods

### Reagents

Triton X-100, sodium acetate, DMSO, DTT (dithiothreitol) and E-64 (trans-epoxysuccinyl-L-leucylamido-(4-guanidino)butane) were purchased from Sigma-Aldrich. The Cruzipain model fluorogenic substrate Z-FR-AMC (benzyloxycarbonyl-phenylalanylarginine-4-methylcoumaryl-7-amide**)** was from Bachem (Bubendorf, Switzerland). Black solid bottom polystyrene Corning® NBS 384-well plates were from Sigma-Aldrich (CLS3654-100EA).

### Cruzipain Purification

Cruzipain (CZP) was purified to homogeneity from epimastigotes of the Tul 2 strain, as described previously^42,43^. Purified Cruzipain was stored at −70 °C as individual aliquots for single use. SDS–PAGE (10%, silver staining) and western blot (Rabbit anti-Cruzipain polyclonal serum) analysis of purified Cruzipain showed the typical double band around 55 kDa and a single band around 38 kDa, corresponding to the complete form, the catalytic domain and C-terminal domain, respectively (Figure S9).

### Anti-kinetoplastid chemical boxes

The HAT and CHAGAS chemical boxes were provided by GlaxoSmithKline. The collection comprised 404 compounds, prepared as 10 mM stock solutions in DMSO (10 µL each) and dispensed in 96 well plates. For primary screening, a working solution (final concentration of 2 mM) for each compound was prepared by 1/5 dilution in DMSO while 1 µL of the 10 mM stock solution was used for secondary screening of selected compounds.

### Cruzipain Assay

Cruzipain activity was assayed fluorimetrically with Z-FR-AMC as substrate in 100 mM acetate buffer pH 5.5 containing 5 mM DTT and 0.01% Triton X-100, as this increased enzyme stability and reduced significantly the number of false positives^31^. Assay was performed in a solid black 384-well plate (final reaction volume ~80 μL) and the release of 7-amino-4-methylcoumarin was monitored continuously at 30 °C with a Beckman Coulter DTX 880 Multimode Reader (Radnor, Pennsylvania, USA) using standard 340 nm excitation and 450 nm emission filter set. Final substrate concentration was set to 2 μM to match previously reported conditions^31^ and being close to the K_M_ value for this substrate^27^. Optimal Cruzipain assay concentration (5.8 nM) was selected from 2-fold serial dilutions to match three criteria: (i) being linearly proportional to V_0_, (ii) display robust signal evolution at 2 μM substrate concentration and (iii) display linear kinetics for enough time to perform several reading cycles (at least 10 cycles, minimum time between cycles: 264 sec) through the 384-wells. In all cases, E-64(final concentration 125 nM) was used as positive inhibition control.

### Assay detection limit and raw estimation of the minimal compound concentration for primary screening

The sensitivity of the established assay to Cruzipain inhibitors was determined by incubation (15 minutes, 30 °C) of the enzyme with decreasing concentrations (10 µM – 0,0625 nM) of E-64 previous to the addition of substrate to initiate reaction. The lowest percentage of inhibition detectable by the assay (%*Inh*^MINIMAL^) was determined as the inhibitor concentration reducing Cruzipain activity to the statistically robust cutoff (µ^C+^ − 3 × SD^C+^) and experimentally determined as 9.87%. Using this information as starting point, we aimed to make a raw estimation of the minimal inhibitor concentration ([*I*]_0_^MINIMAL^) required to achieve %*Inh*^MINIMAL^ to stablish a reasonable initial compound concentration for primary screening. For any reversible Cruzipain inhibitor, the equilibrium (E + I ↔ E − I) is governed by the law of mass action. For simplicity of this preliminary calculation, we omitted the effect of initial substrate concentration on this equilibrium and substituted the true value of Ki by the apparent term Ki^app^ (Eq. 1). The general principle of mass conservation can be formalized as Eq. 2 and Eq. 3 for Enzyme- and Inhibitor-associated species, respectively. Finally, Eq. 4 describes %*Inh* as a function of the complex [*E* − *I*] and initial enzyme concentration [*E*]_0._

1 $${{\rm{Ki}}}^{{\rm{app}}}=[E]\cdot [I]/[E-I]$$

2 $${[E]}_{0}=[E]+[E-I]$$

3 $${[I]}_{0}=[I]+[E-I]$$

4 $$ \% Inh=100\cdot [E-I]/{[E]}_{0}$$

Integration of these four equations permitted to define Ki^app^ as a function of %*Inh*, [*E*]_0_ and [*I*]_0_. In the particular case of %*Inh* = %*Inh*^MINIMAL^; [*I*]_0_ became [*I*]_0_^MINIMAL^:

5 $${{\rm{Ki}}}^{{\rm{app}}}=(\frac{100- \% In{h}^{{\rm{MINIMAL}}}}{ \% In{h}^{{\rm{MINIMAL}}}})\cdot ({[I]}_{0}^{{\rm{MINIMAL}}}-\frac{ \% In{h}^{{\rm{MINIMAL}}}.{[E]}_{0}}{100})$$

In practice, the value of [*I*]_0_^MINIMAL^ underestimate the true compound concentration required to achieve Cruzipain inhibition of 9,87%, given the presence of [*S*]_0_ ~ K_M_ and that many active compounds are expected to act as competitive inhibitors.

### Primary screening

To perform the high-throughput screening, 1 μL of each compound (2 mM in DMSO), E-64 (10 μM in DMSO) or DMSO (negative controls) were dispensed into 384-well Corning black solid-bottom assay plates. Then, 40 μL of 100 mM NaAc, 5 mM DTT, 0.01% Triton X-100 pH 5.5 containing Cruzipain (11,6 nM) were added to each well, plates were homogenized (30 seg, orbital, medium intensity) and each well subjected to a single autofluorescence read (exc/ems = 340/450 nm). Plates were incubated in darkness for 15 min at 30 °C and then 40 μL of Z-FR-AMC (4 μM) in assay buffer were added to each well to start the reaction. After homogenization (30 seg, orbital, medium intensity), the fluorescence of AMC (exc/ems = 340/450 nm) was acquired kinetically for each well (12 read cycles, one cycle every 300 seconds).

Raw screening measurements were used to determine the slope (dF/dt) of progression curves by linear regression for control and compound wells. In the case of control-dependent hit selection criterium, percent inhibition (%*Inh*) was calculated for each compound according to Eq. 6:

6 $$ \% Inh=100\cdot [1-\frac{({\rm{dF}}/{{\rm{dt}}}^{{\rm{WELL}}}-{\mu }^{{\rm{C}}-})}{({\mu }^{{\rm{C}}+}-{\mu }^{{\rm{C}}-})}]$$

where dF/dt^WELL^ represents the slope of each compound well and µ^C+^ and µ^C−^ the average of Cruzipain (no-inhibition) and substrate (no-enzyme) controls, respectively.

### Secondary assay

Fifteen compounds selected from primary screening were re-tested in a dose-response manner (final concentration ranging from 62.5 μM to 7.5 pM) using identical assay conditions, except for higher Cruzipain concentration (final concentration 11.6 nM) to facilitate the identification of tight-binding inhibitors. To avoid any positional and/or association bias, we randomly defined the row position for each compound. One μL of compounds stock (10 mM in DMSO) and E-64 (10 mM in DMSO) were added to the first well of column 1, followed by addition of 40 μL of 100 mM NaAc, 5 mM DTT, 0.01% Triton X-100 pH 5.5 buffer. After addition of 20 μL of the same buffer to subsequent wells of the plate, 22 serial 2-fold dilutions were made horizontally. The last two positions of every row were used, alternatively, for C^+^ and C^−^ controls to reduce any positional and/or association bias. Then, 20 μL of activity buffer containing Cruzipain (46.4 nM) were added to each well, except for those corresponding to C^−^; completed with 20 μL of activity buffer. After homogenization, 15 minutes of incubation at 30 °C and autofluorescence measurement, 40 μL of a 4 μM solution of Z-FR-AMC substrate (in activity buffer) was added to the previous mix. Data collection and processing were performed exactly as described above. Percentage of Cruzipain residual activity was calculated for each condition according to Eq. 7:

7 $$ \% Res.Act{.}^{CRUZIPAIN}=100\cdot [\frac{({\rm{dF}}/{{\rm{dt}}}^{{\rm{WELL}}}-{\mu }^{{\rm{C}}-})}{({\mu }^{{\rm{C}}+}-{\mu }^{{\rm{C}}-})}]$$

where dF/dt^WELL^ represents the slope of each compound well and µ^C+^ and µ^C−^ the average of Cruzipain (no-inhibition) and substrate (no-enzyme) controls, respectively. The IC_50_ and Hill slope parameters for each compound were estimated by fitting the four-parameter Hill equation to experimental data from dose-response curves using the GraphPad Prism program (version 5.03).

### Substructure search

Substructure searches were performed directly against a complete SDF file containing all bioactive compounds in ChEMBL (Release 22.1)^23^, using the OpenBabel substructure search functionality. Prior to substructure searches, an index was created using the OpenBabel indexing tools, to accelerate searches. Substructures (fragments) were automatically obtained using MolBlocks^30^ to allow for a standardized fragmentation strategy. Compounds found to be a superstructure in each substructure search were then checked for single-target reported bioactivities. These were directly retrieved from a CHEMBL MySQL database local server installation using a custom Perl script, filtering results to those compounds that had been tested and proven active against at least one specific target. All compounds were then grouped according to the protein family of the assayed targets and considered “related” when the target protein family was a protease. For ratio calculation, the amount of related bioactivities was divided by the total amount of single target bioactivities, like

$${\rm{Related}}\,{\rm{Bioactivity}}\,{\rm{Ratio}}=\frac{{\rm{Related}}\,{\rm{Bioactivities}}\,{\rm{reported}}}{{\rm{All}}\,{\rm{single}}\,{\rm{target}}\,{\rm{Bioactivities}}\,{\rm{reported}}}\times 100$$

### Tanimoto similarity searches

Lists of compounds in GSK chemical boxes were obtained from Peña *et al*.^22^. Compound structures (MDL files) and molecule synonyms were then retrieved from the ChEMBL database (Release 22.1)^23^ using their GSK names as identifiers in an automated compound mapping process. All database operations were performed on a local server using MySQL. With the complete list of compounds and their structures available, an ALL vs ALL fingerprint-based Tanimoto search was done using both FP2 and FP4 OpenBabel indexes. Results for both fingerprints were combined to obtain a weighted fingerprint. Weighted matrix was finally used to create an ALL vs ALL matrix. The following equation was used to weight the results:

$${{\rm{T}}}_{{\rm{index}}}=\frac{2\cdot {\rm{FP}}{2}_{{\rm{index}}}+8\cdot {\rm{FP}}{4}_{{\rm{index}}}}{10}$$

To facilitate visualization of tanimoto scores when summarizing large chemical similarity searches, we normalized tanimoto scores using the following sigmoid transformation. (see e.g. Figure S5).

$${\rm{f}}({{\rm{T}}}_{{\rm{i}}{\rm{n}}{\rm{d}}{\rm{e}}{\rm{x}}})=\frac{1}{1+{{\rm{e}}}^{-b({\rm{T}}{\rm{i}}{\rm{n}}{\rm{d}}{\rm{e}}{\rm{x}}-0.4)}}$$

The transformation minimizes lower tanimoto scores (e.g. those <0.4) and gives a boost to medium tanimoto scores (e.g: those greater than 0.4 but lower than 0.8). This allowed to get an easier way to visualize compounds that may result interesting, while minimizing the impact on those compounds for which the tanimoto index was below threshold (0.4).

### Docking experiments

To prepare for covalent docking to Cruzipain (PDB code 3IUT), the protein setup was performed using the Protein Preparation Wizard (ref.^1^) using default parameters, and ligands TCMDC-143393 and TCMDC-143390 were prepared using default parameters in LigPrep^44^, both in Schrödinger Suite^45^. The CovDock algorithm^34^ was used to covalently dock the ligands, specified with the attachment point of Cys25 in a 10 Å × 10 Å × 10 Å box using a nucleophilic addition to a nitrile reaction scheme. Briefly, the algorithm works by: 1) Mutating the covalent attachment point residue to alanine and using Glide^46^ to dock and pre-position the non-reacted ligand correctly in the binding site; 2) Mutating the attaching residue back and sampling its rotameric states with Prime^47^; 3) Forming the specified covalent bond type to the ligand pose; 4) Minimizing, clustering, scoring, and ranking the poses using GlideScore. The top 10 poses of each ligand were retained and compared visually to known cruzain-inhibitor complexes.

### Data Availability

The datasets generated during and/or analysed during the current study are available from the corresponding author on reasonable request.

## Electronic supplementary material

Supplementary Information
