# Author Correction: Novel scaffolds for inhibition of Cruzipain identified from high-throughput screening of anti-kinetoplastid chemical boxes

**DOI:** 10.1038/s41598-018-26961-w

**Published:** 2018-06-04

**Authors:** Emir Salas-Sarduy, Lionel Urán Landaburu, Joel Karpiak, Kevin P. Madauss, Juan José Cazzulo, Fernán Agüero, Vanina Eder Alvarez

**Affiliations:** 1 Instituto de Investigaciones Biotecnológicas – Instituto Tecnológico de Chascomús, Universidad Nacional de San Martín – CONICET, San Martin, B1650HMP Buenos Aires Argentina; 2 GlaxoSmithKline R&D, Molecular Design US, Pennsylvania, Upper Providence PA USA; 3 GlaxoSmithKline R&D, Trust in Science, Pennsylvania, Upper Providence PA USA

Correction to: *Scientific Reports* 10.1038/s41598-017-12170-4, published online 21 September 2017

The original version of this Article contained a typographical error in the spelling of the author Joel Karpiak, which was incorrectly given as Joel X. Karpiak. This has now been corrected in the PDF and HTML versions of the Article and in the accompanying Supplementary Information file.

