# Supplementary material for: Novel scaffolds for inhibition of Cruzipain identified from high-throughput screening of anti-kinetoplastid chemical boxes
Source: Sci Rep. 2017 Sep 21;7:12073. doi: 10.1038/s41598-017-12170-4 (PMC5608908; doi:10.1038/s41598-017-12170-4)
Supplement: Supplementary file 1 — Supplementary Information [file 41598_2017_12170_MOESM1_ESM.pdf]

**Electronic supplementary material to "** Novel scaffolds for inhibition of Cruzipain identified from high-throughput screening of anti-kinetoplastid chemical boxes"

**Authors:** Emir Salas-Surduy[1], Lionel Urán Landaburu[1], Joel Karpiak[2], Kevin P. Madauss[3], Juan José Cazzulo[1], Fernán Agüero[1]* and Vanina Eder Alvarez[1]*

**Affiliations:** [1] Instituto de Investigaciones Biotecnológicas – Instituto Tecnológico de Chascomús, Universidad Nacional de San Martín – CONICET, San Martin, B1650HMP, Buenos Aires, Argentina

[2] GlaxoSmithKline R&D, Molecular Design US, Upper Providence PA

[3] GlaxoSmithKline R&D, Trust in Science, Upper Providence PA

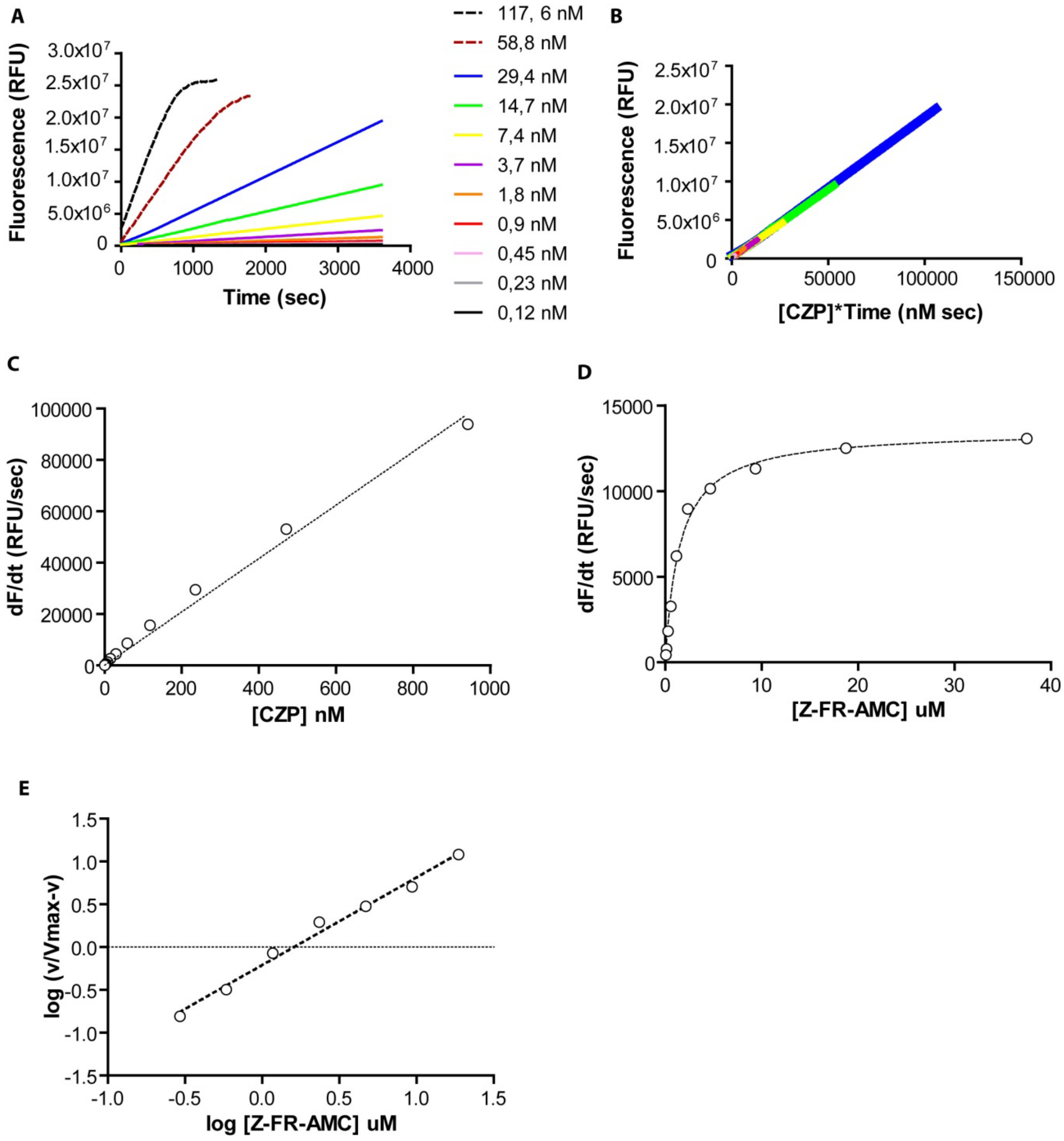


**Figure S1. Continuous fluorogenic assay for Cruzipain.** A) Kinetic progression curves for different Cruzipain concentrations and fixed substrate concentration (2 μM). B) Selwyn test for different Cruzipain concentrations (substrate concentration: 2 μM). C) Curve of $V_0$ vs. $[E]_0$ for Cruzipain. D) Michaelis-Menten plot for Cruzipain (5.8 nM). E) Hill plot for Cruzipain (5.8 nM).

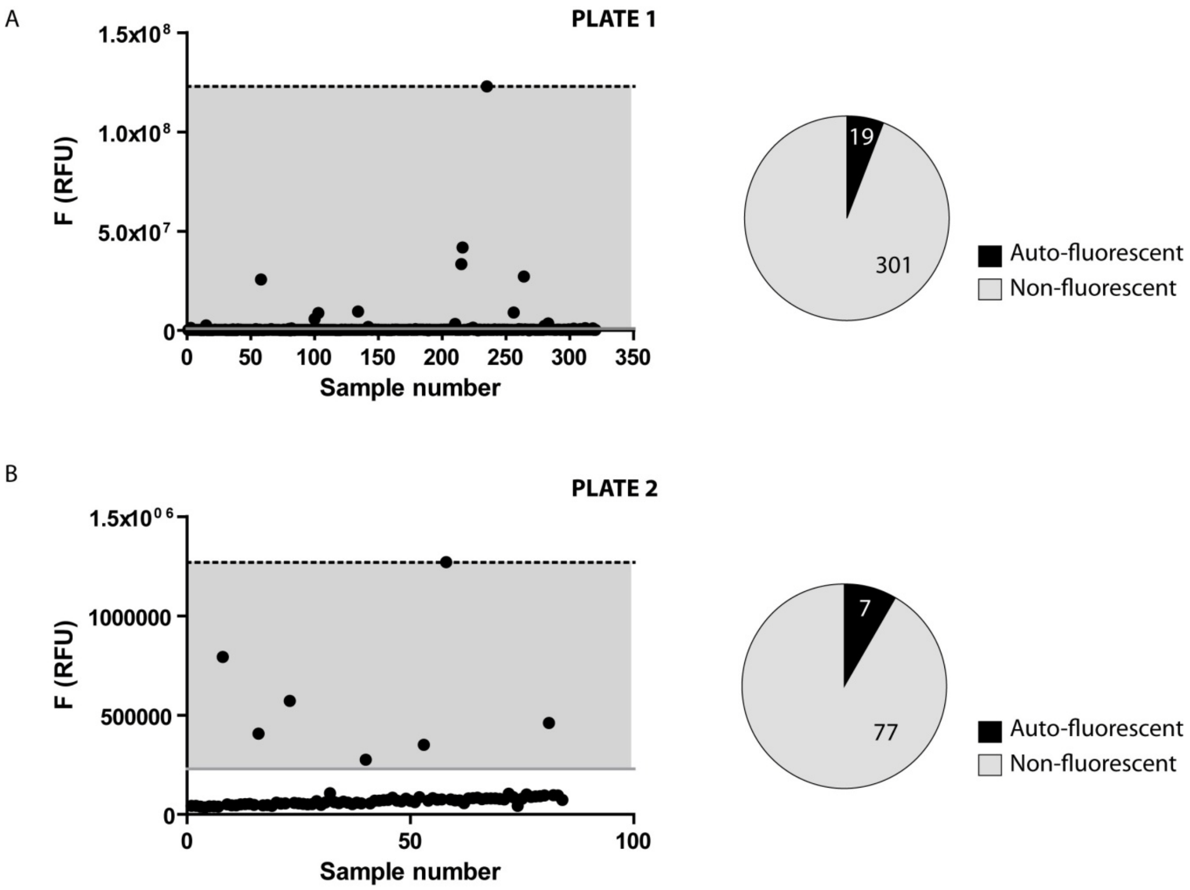


**Figure S2. Autofluorescence for compounds of HAT and Chagas chemical boxes under assay conditions (λexc/emss=355nm/460nm).** Those compounds showing auto-fluorescence readouts equal or higher than 1/3 of the total Fluorescence increase of AMC during the 60 minutes assay (equivalent to 25% of total assay fluorescence) were considered autofluorescent. Dashed line in each graphic represents the arbitrary cutoff established. A) Distribution of auto-fluorescent readouts for compounds in Plate 1 B) Distribution of auto-fluorescent readouts for compounds in Plate 2.

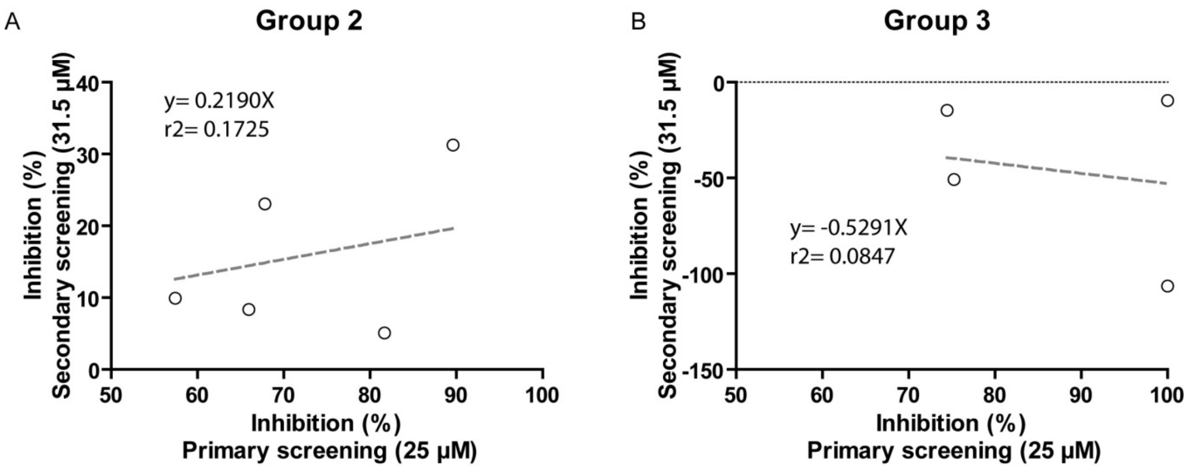


**Figure S3. Compounds showing non-reproducible Cruzipain inhibition.** A) Group 2: Compounds showing no apparent correlation in Cruzipain inhibition between primary and secondary screenings at an equivalent compound concentration. B) Group 3: Compounds showing a different phenotype (activation instead of Cruzipain inhibition) during secondary screening at an equivalent compound concentration.

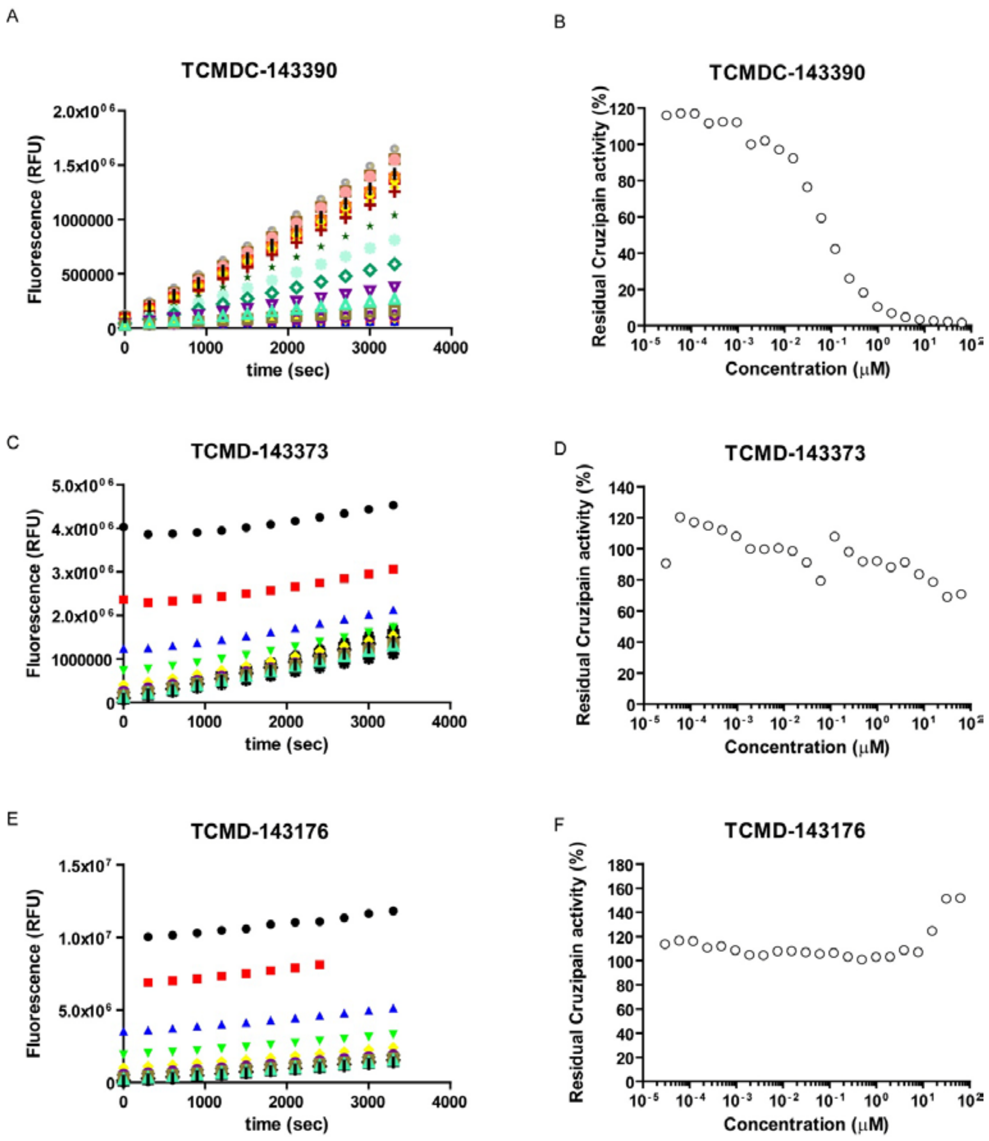


**Figure S4. Highly auto-fluorescent compounds caused artefactual (non-typical) dose-response curves.** A) Collection of progression curves for TCMDC-143390, a representative compound from group 1 (each curve corresponds to a different compound concentration ranging from 62.5 μM – 7.5 pM). B) Dose-response curve for TCMDC-143390 showing the typical behavior. C) Collection of progression curves starting at very different Fluorescence values for TCMDC-143373, a representative compound from Group 2 (each curve corresponds to a different compound concentration ranging from 62.5 μM – 7.5 pM). D) Dose-response curve for TCMDC-143373 showing an erratic behavior. E) Collection of progression curves starting at very different Fluorescence values for TCMDC-143176, a representative compound from Group 3 (each curve corresponds to a different compound concentration ranging from 62.5 μM – 7.5 pM). F) Dose-response curve for TCMDC-143176 showing an activation phenotype at highest concentrations.

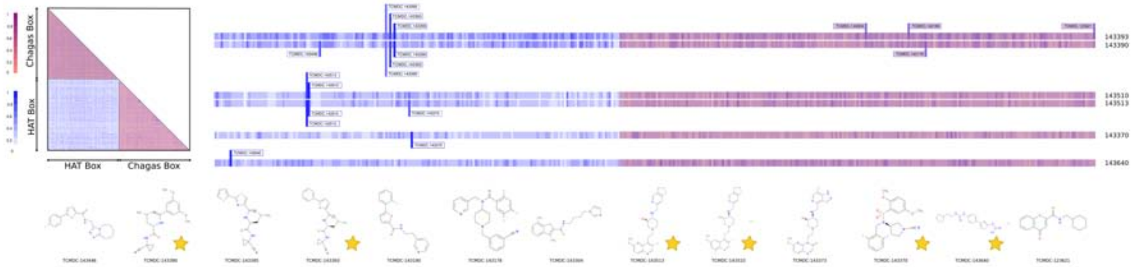


**Figure S5. Chemical similarity across GSK Chagas and HAT boxes.** An ALL vs ALL chemical similarity analysis was conducted using compounds from both Chagas and HAT chemical boxes. Both type 2 and type 4 fingerprints (OpenBabel) were calculated and combined to build a heatmap visualization for the similarity matrix (left). A zoom-in for the lead compounds is shown to focus on highly similar compounds that could have been ignored during activity screening. Hits found were neither significantly active nor too related structurally to the lead compounds.

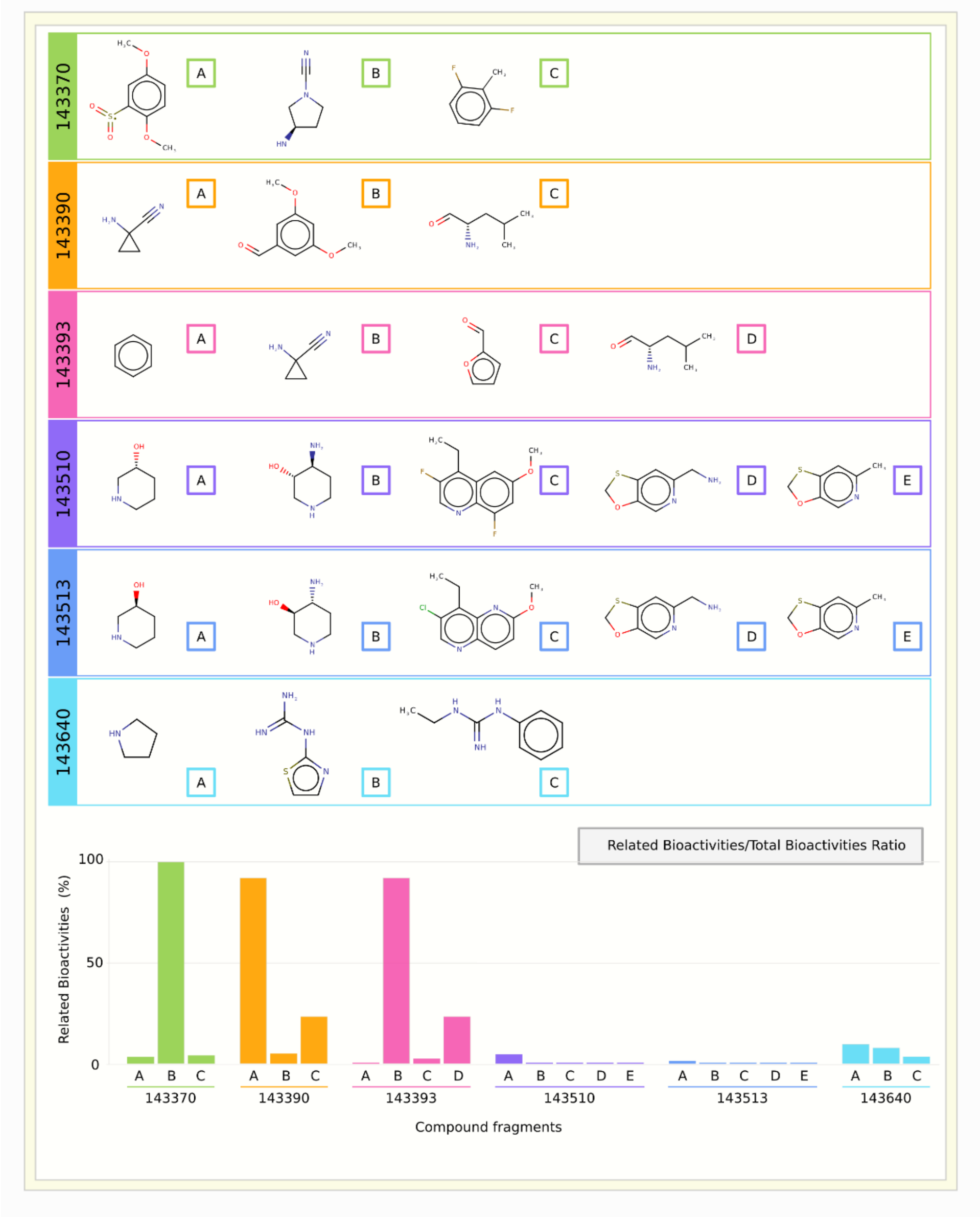


**Figure S6. Complete list of MolBlocks (substructure fragments) for each of the Cruzipain hits identified in this work.** In the bottom panel, the enrichments (bioactivity ratios) in protease-associated bioactivities are shown using the same bar chart as in Figure 6.

| Lead Compounds | | Molecule Fragments | HITS | Bioactivities | Single Protein Bioactivities | Related Bioactivites | Ratio (%) |
|---|---|---|---|---|---|---|---|
| **TCMDC-143370** | **A** | COc1ccc(c(c1)[S](=O)=O)OC | 442 | 3948 | 2441 | 85 | |
| | **B** | **N#CN1CC[C@H](C1)N (SS2)** | 23 | 171 | 66 | 66 | |
| | **C** | Cc1c(F)cccc1F | 5627 | 33004 | 13513 | 566 | |
| **TCMDC-143390** | **A** | **N#CC1(N)CC1 (SS1)** | 374 | 1952 | 963 | 887 | |
| | **B** | COc1cc(OC)cc(c1)C=O | 5203 | 42909 | 13184 | 674 | |
| | **C** | O=C[C@H](CC(C)C)N | 30112 | 160067 | 55476 | 12988 | |
| **TCMDC-143393** | **A** | c1ccccc1 | * | * | * | * | |
| | **B** | **N#CC1(N)CC1 (SS1)** | 374 | 1952 | 963 | 887 | |
| | **C** | O=Cc1ccco1 | 19619 | 194304 | 110536 | 2864 | |
| | **D** | O=C[C@H](CC(C)C)N | 30112 | 160067 | 55476 | 12988 | |
| **TCMDC-143510** | **A** | O[C@@H]1CCCNC1 | 5095 | 38701 | 17042 | 810 | |
| | **B** | CCc1c(F)cnc2c1cc(OC)cc2F | 6 | 0 | 0 | 0 | |
| | **C** | NCc1ncc2c(c1)SCO2 | 13 | 45 | 3 | 0 | |
| | **D** | N[C@H]1CCNC[C@@H]1O | 320 | 1475 | 756 | 4 | |
| | **E** | Cc1ncc2c(c1)SCO2 | 14 | 45 | 3 | 0 | |
| **TCMDC-143513** | **A** | O[C@H]1CCCNC1 | 96 | 1475 | 857 | 12 | |
| | **B** | CCc1c(Cl)cnc2c1nc(OC)cc2 | 14 | 44 | 4 | 0 | |
| | **C** | NCc1ncc2c(c1)SCO2 | 13 | 45 | 3 | 0 | |
| | **D** | N[C@@H]1CCNC[C@H]1O | 320 | 1475 | 756 | 4 | |
| | **E** | Cc1ncc2c(c1)SCO2 | 14 | 45 | 3 | 0 | |
| **TCMDC-143640** | **A** | C1CCCN1 | 125113 | 750374 | 289146 | 27944 | |
| | **B** | NC(=N)Nc1nccs1 | 433 | 3119 | 596 | 47 | |
| | **C** | CCNC(=N)Nc1ccccc1 | 2818 | 21279 | 5581 | 196 | |

**Figure S7. Table of bioactivity hits for each molblock/fragment**. Each substructure fragment was used as query against ChEMBL. Identified bioactivities were classified as ‘related’ (target is a protease) or non-related. These figures were used for the ratio calculations shown in Figures 6 and S6. This ratio therefore represents a quick indicator to point out which substructures could be related to a protease inhibitor scaffold.

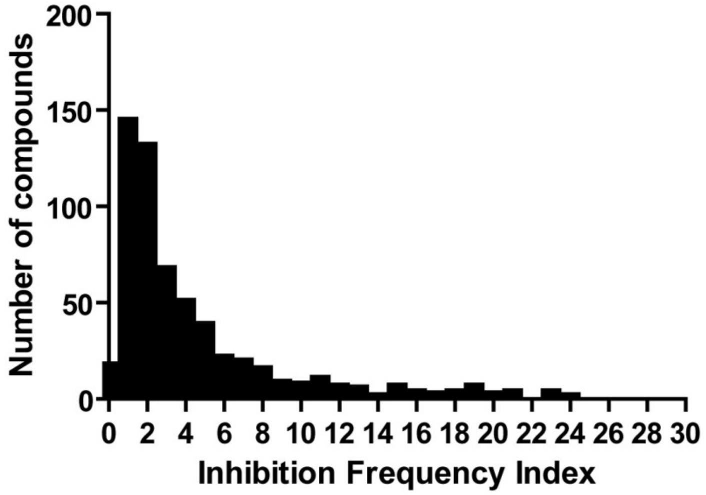


**Figure S8. Distribution of the Inhibition Frequency Index parameter among the compounds in the anti-kinetoplastid boxes.** Inhibition frequency index: relative frequency with which a compound has scored more than 50% inhibition in an HTS assay.

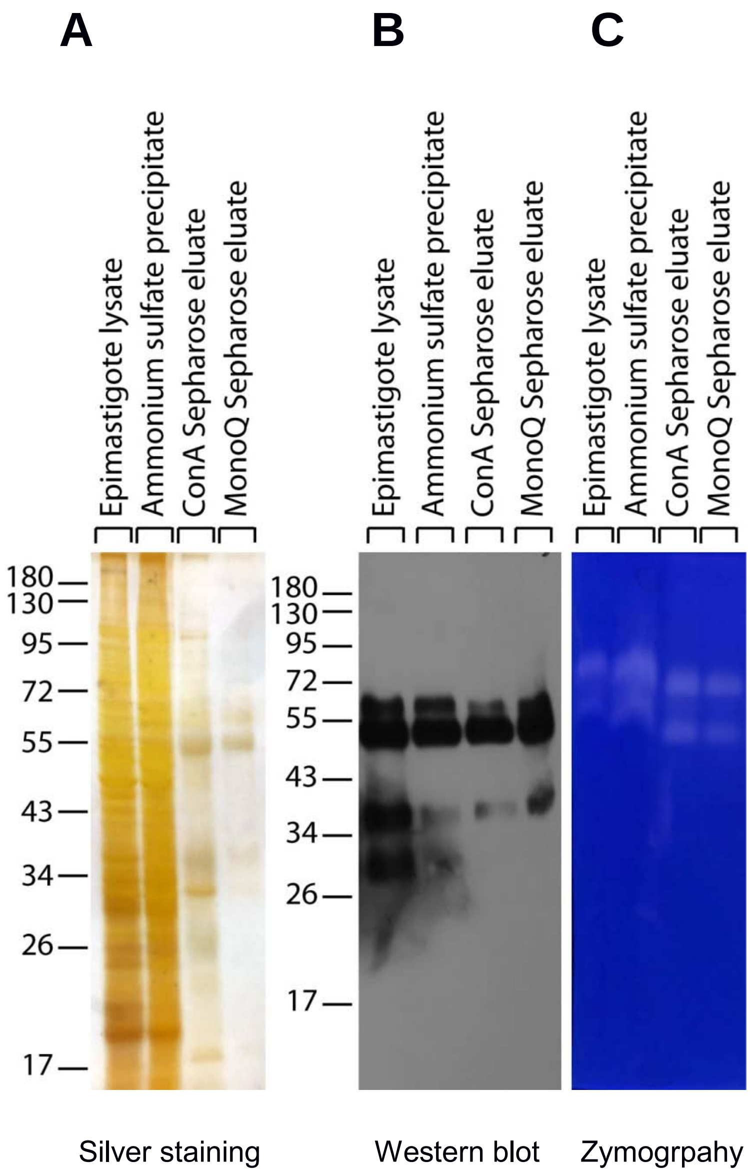


**Figure S9. Purification of endogenous Cruzipain from *T. cruzi* epimastigotes.** Enzyme was purified by a combination of ammonium sulfate precipitation, affinity chromatography on Con A-Sepharose and anion exchange chromatography on a MonoQ column. A) SDS-PAGE (silver staining) analysis of resultant fractions from the purification steps. B) Western-Blot (anti-Cruzipain specific rabbit polyclonal sera) analysis of resultant fractions from the purification steps. C) Zymography (gelatin as incorporated substrate) analysis of resultant fractions from the purification steps.
